# Boosting neuraminidase immunity in the presence of hemagglutinin with the next generation of influenza vaccines

**DOI:** 10.1038/s41541-024-01011-x

**Published:** 2024-11-19

**Authors:** Guadalupe Cortés, Irina Ustyugova, Timothy Farrell, Clint McDaniel, Colleen Britain, Christopher Romano, Siré N’Diaye, Lingyi Zheng, Mithila Ferdous, Justin Iampietro, Svetlana Pougatcheva, Lauren La Rue, Liqun Han, Fuqin Ma, Svetlana Stegalkina, Satyajit Ray, Jianxin Zhang, Mario Barro

**Affiliations:** 1 Vaccines R&D, Sanofi, Cambridge, MA USA; 2 Yoh Services LLC, Philadelphia, PA USA; 3 Vaccines R&D, Sanofi, Swiftwater, PA USA

**Keywords:** Drug development, Outcomes research

## Abstract

Neuraminidase (NA), the second most abundant surface glycoprotein on the influenza virus, plays a key role in viral replication and propagation. Despite growing evidence showing that NA-specific antibodies correlate with resistance to disease in humans, current licensed vaccines focus almost entirely on the hemagglutinin (HA) antigen. Here, we demonstrate that recombinant NA (rNA) protein is highly immunogenic in both naïve mice and ferrets, as well as in pre-immune ferrets, irrespective of the level of match with preexisting immunity. Ferrets vaccinated with rNA developed mild influenza disease symptoms upon challenge with human H3N2 influenza virus, and anti-NA antibody responses appeared correlated with reduction in disease severity. The addition of rNA to a quadrivalent HA-based vaccine induced robust NA-specific humoral immunity in ferrets, while retaining the ability to induce HA-specific immunity. These results demonstrate that the addition of rNA is a viable option to increase immunogenicity and potentially efficacy versus currently licensed influenza vaccines by means of boosting NA immunity.

## Introduction

Influenza is a highly contagious viral infection. Seasonal disease epidemics, caused predominantly by influenza types A and B, occur every winter in temperate regions. The disease persists as a considerable global public health concern, responsible for an estimated 3–5 million severe cases and up to 650,000 deaths annually^1^. Those with chronic underlying medical conditions are at increased risk of severe influenza illness and/or complications caused by exacerbation of their underlying condition, resulting in a disproportional burden of influenza-related illness in these at-risk groups^2^. Nonetheless, life-threatening complications or even death, may occur in otherwise healthy individuals, particularly among infants (aged <1 year) and the elderly (aged ≥65 years)^3^. Annual influenza vaccination of at-risk populations is the most effective disease management strategy and can help prevent overburdening healthcare systems during epidemics. Vaccination also provides protection beyond prevention of influenza infection and disease by reducing complications, such as pneumonia and cardiorespiratory-associated hospitalizations, particularly among infants and the elderly^4^.

Coronavirus disease 2019 (COVID-19)—the pandemic caused by severe acute respiratory syndrome coronavirus 2 (SARS-CoV-2)—has had unparalleled impact on global communicable diseases morbidity and mortality. Wide-spread introduction of COVID-19 containment measures such as mandated social distancing and shielding in most countries appeared to reduce the transmission of some communicable diseases^5^. However, the excess deaths observed during COVID-19 in some countries were comparable to those reported during severe influenza epidemics^6^. Of concern, the data available suggested that SARS-CoV-2 and influenza co-infection may be associated with increased severity and mortality^7^.

The influenza virus surface proteins, hemagglutinin (HA) and neuraminidase (NA), have critical roles in virus propagation and are important targets for the host immune system^8^, making them key candidates for prophylactic influenza vaccines. Antibodies targeting these two surface proteins inhibit viral attachment/fusion and release, respectively, effectively neutralizing the influenza virus. However, influenza viruses acquire mutations in their HA and NA proteins on a regular basis enabling them to evade pre-existing immunity. Thus, seasonal influenza vaccine efficacy may be highly variable due to strain match/mismatch and waning immunity over time. Annual seasonal influenza vaccinations are recommended, but may require yearly reformulation to match changes in circulating strains identified through surveillance.

Current licensed influenza vaccines predominantly focus on HA immunity, with standardized antigen content, but with no such requirement for NA content. Thus, the latter can vary by vaccine and manufacturing process, and the exact breadth of NA-based protection remains unknown^9,10^. Despite evidence that NA antibodies are protective and broadly cross-reactive, and that NA represents a key target for the only approved influenza antiviral treatments, there is a paucity of data on the robustness of NA antibody responses. Furthermore, concerns about immunodominance of HA over NA have been raised^8,11^. Emerging evidence suggests that NA can induce protective antibodies that often exhibit increased breadth of recognition across strains^10^. Indeed, studies in humans suggest NA inhibition (NAI) antibody titers are independently correlated with protection, and may even be more predictive of protection or reduced disease than HA inhibition (HAI) antibody titers^12–15^.

Influenza NA, a homotetrameric type II transmembrane glycoprotein, consists of a globular head domain, a central stalk with a hydrophobic transmembrane domain, and a short, N-terminal cytoplasmic domain^16^. Tetramerization of the head domain is required for both enzymatic activity^17^ and induction of protective antibody responses^18^. Differences in stalk length between influenza A virus subtypes may^19^, in part, influence enzymatic activity as it affects NA protrusion above the viral envelope relative to HA^20,21^. Nonetheless, irrespective of variation in NA stalk regions between different influenza A virus subtypes, NAs stalks share common structural features, including a potential glycosylation site and at least one cysteine residue^21^. These two features are thought to contribute to tetramer stabilization, the latter through formation of disulfide bonds. The transmembrane domain, which anchors NA to the viral envelope, may also assist in formation of the tetrameric conformation^22^.

Vaccines targeting inclusion of optimal NA content represent an attractive option as they would be expected to increase protection compared to conventional influenza vaccines^23^. Moreover, NA tends to evolve independently and at a slower pace in circulating strains than HA, and as such, would be particularly advantageous in seasons where antigenic changes (either drift or shift) in HA occur. Recent preclinical data suggest that targeting both HA and NA would lead to better antiviral activity and protection than HA alone, and would likely compensate for reduced vaccine efficacy caused by HA drift and resultant HA mismatch in vaccine formulations^24–27^. The inclusion of recombinant soluble NAs may have several advantages; they can be produced in large quantities, in a relatively short time, and with the optimal tetrameric conformation. Recovery of correctly folded tetrameric NA is important for effective immunological activity. The use of exogenous tetramerization domains fused to the NA head region enables preparation of functional recombinant soluble tetrameric NA^17,22,28^.

In this study, we produced soluble recombinant NAs from a number of subtypes by fusion of the globular head domain with the tetrabrachion tetramerization domain (rTET-NA), shown previously to enable formation of stable functional tetrameric NA^29^, and characterized their immunogenicity in both naïve mice and ferrets. In ferrets, we additionally demonstrated rTET-NA immunogenicity irrespective of pre-immune status or presence of HA, and protection against challenge with human H3N2 influenza virus. Moreover, the addition of rTET-NA to a quadrivalent HA-based influenza vaccine induced robust NA-specific humoral immunity while retaining the ability to induce HA-specific immunity.

## Results

### Expression, purification and characterization of rTET-NA

The rTET-NA constructs derived from NAs across the two influenza A virus subtypes and two influenza B virus lineages present in the WHO recommended influenza vaccine composition for the 2018-2019 Northern hemisphere influenza season (A/Michigan/45/2015 N1; A/Singapore/INFIMH-16-0019/2016 N2; B/Colorado/06/2017 B/Victoria/2/87-like lineage; and B/Phuket/3073/2013 B/Yamagata/16/88-like lineage) were expressed in Chinese hamster ovary-S (CHO-S) cells and purified for further characterization.

**Fig. 1:**
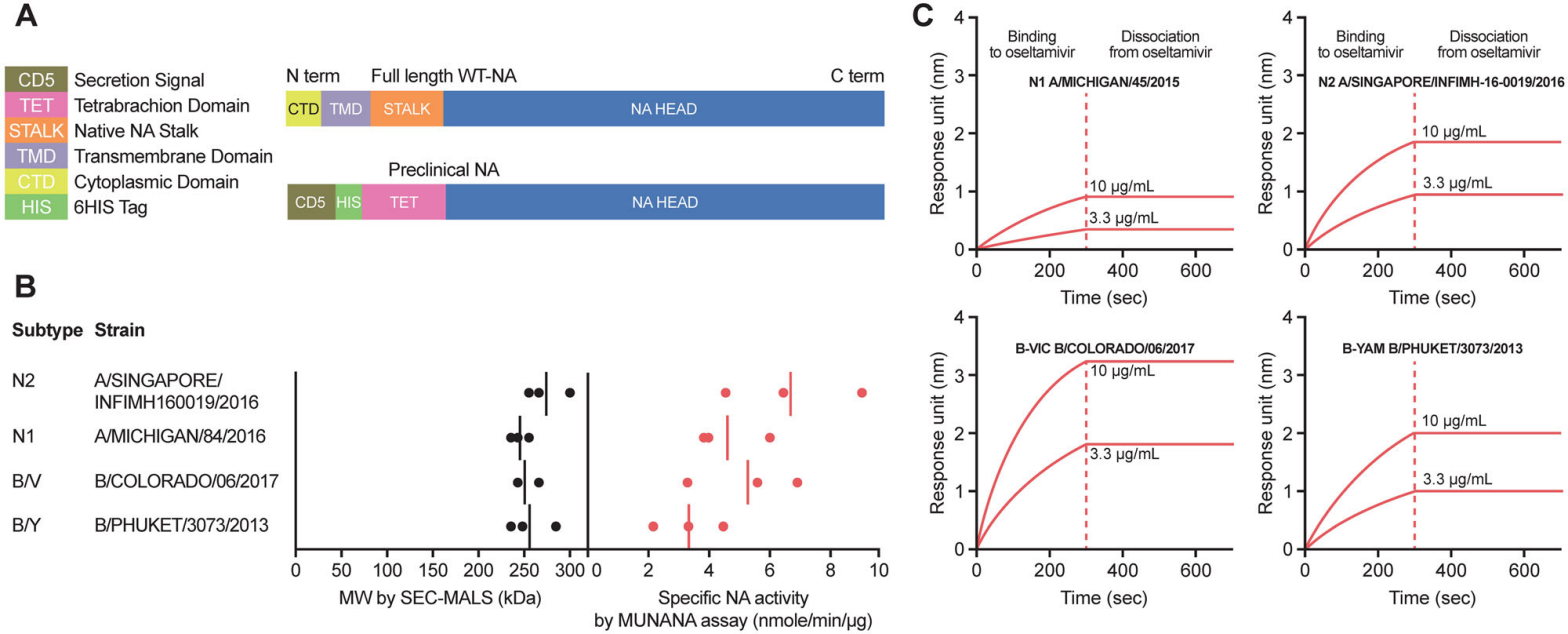
Structural analysis of rTET-NA constructs. **A** Diagram of recombinant tetrabrachion neuraminidase (rTET-NA) compared to the full-length NA. **B** Molecular weight determined by SEC-MALS and enzymatic activity of four different influenza virus strain rTET-NAs (dots represent independent experiments and solid lines represent the average measurement). **C** Oseltamivir-binding kinetics analysis for four different influenza virus strain rTET-NAs.

A schematic representation of rTET-NA derived from A/Singapore/INFIMH-16-0019/2016 N2 is shown in Fig. 1A. Full-length wild-type influenza virus neuraminidase includes a short, N-terminal cytoplasmic domain, a transmembrane region, a stalk region and a head region. In rTET-NA, the cytoplasmic domain, the transmembrane region, and most of the stalk region are replaced by a ‘secretion signal’ peptide, a histidine-tag, and a heterologous tetrabrachion tetramerization domain^29^. Clear tetramerization of rTET-NAs was demonstrated by size exclusion chromatography with multi-angle light scattering (SEC-MALS), as well as indirectly through NA enzymatic activity with the 2′-(4-methylumbelliferyl)-α-d-N-acetylneuraminic acid (MUNANA) assay and binding to oseltamivir-phosphate, both of which necessitate rTET-NA tetramer formation (Fig. 1B and C). SEC-MALS showed that all rTET-NAs are formed as tetramers with molecular mass ( ~ 250 kDa) about four-fold greater than that expected for monomers ( ~ 64 kDa). rTET-NA enzymatic activity ranged from 4 to 9 nmol/min/μg across the four influenza virus strains assessed as determined with the MUNANA assay. All four rTET-NAs from the influenza virus strains were able to bind to oseltamivir-phosphate with high affinity and did not dissociate under the conditions assessed.

### rTET-NA immunogenicity in mice

**Fig. 2:**
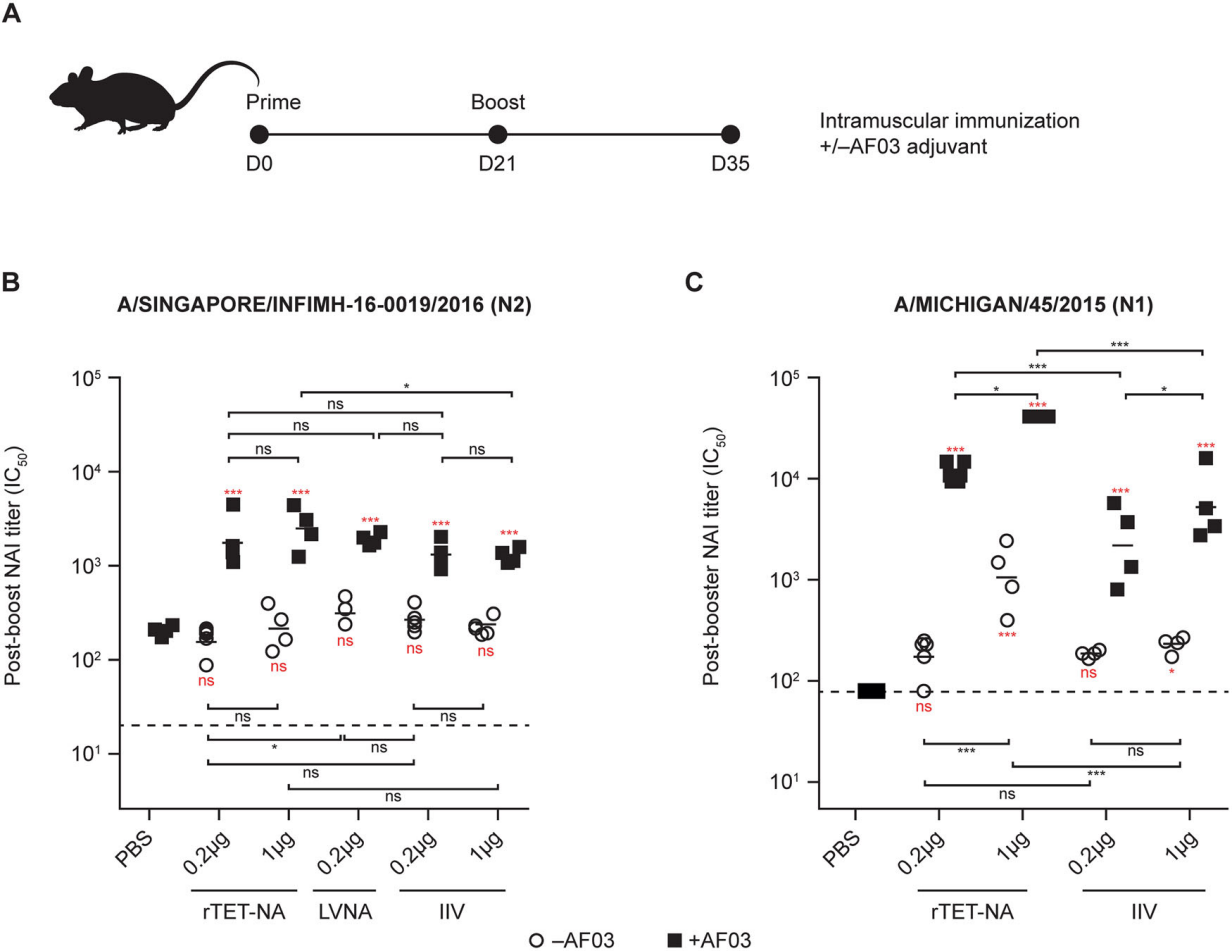
Immunogenicity of A/Singapore/INFIMH-16-0019/2016 N2 and A/Michigan/45/2015 N1 rTET-NAs, LVNA and IIV preparations in naïve mice. Female BALB/c mice (*n* = 8 per group) were immunized twice (intramuscularly) and terminally bled 2 weeks after second immunization (**A**). Sera were collected two weeks after booster vaccination; sera pools from two animals were created and in turn tested via ELLA to assess NAI antibody activity using reassortant H6N2 A/Singapore/INFIMH-16-0019/2016 (**B**) or H6N1 A/Michigan/45/2015 (**C**) viruses as sources of sialidase, respectively. º symbol represents IC_50_ NAI titers without AF03 addition and ■ represents groups with AF03 addition. The solid lines represent the average measurement, and the dashed lines indicate the starting serum dilution used for testing. *p < 0.05; **p < 0.01; ***p < 0.001 (the red text/symbols are comparisons vs adjuvanted PBS/control). *p* < 0.001 for all comparisons between adjuvant and non-adjuvanted vaccines at same dose level.

We initially used a naïve mouse model to assess the immunogenicity of rTET-NA (N1 and N2 subtypes) compared to virus-derived full-length NA. Mice were vaccinated twice (21 days apart) with 0.2 μg or 1 μg of N2 rTET-NA derived from A/Singapore/INFIMH-16-0019/2016, N1 rTET-NA derived from A/Michigan/45/2015, or monovalent inactivated influenza vaccine (IIV; N1 and N2 subtypes), or 0.2 μg live virus-derived NA (LVNA; N2 subtype only) with or without AF03 adjuvant containing 5% squalene. NAI antibody titers were measured in sera two weeks after the last dose. For all adjuvanted NA-containing preparations, including rTET-NA, NAI antibody responses were detectable at significantly higher titers than adjuvanted phosphate buffer saline (PBS) control, while only N1 rTET-NA at 1 μg dose showed significant increase over PBS without adjuvant (Fig. 2). Similar effect of adjuvant was obtained for NA enzyme-linked immunosorbent assay (ELISA), though in contrast to NAI, NA ELISA titers were significantly higher than adjuvanted PBS even for unadjuvanted vaccines, irrespective of subtype (N1, N2) (Fig. S1). The immunogenicity of adjuvanted rTET-NA from the N2 subtype was comparable to other NA-containing viral preparations (LVNA and IIV) for most doses, with the exception of 1 μg dose of adjuvanted rTET-NA that was significantly higher than IIV, while that from the N1 subtype was significantly higher than IIV for the same dose when adjuvanted at 0.2 and 1 μg dose and without adjuvant at 1 μg dose. It is possible that this subtype-specific difference may be due to the split inactivation process for IIV, potentially resulting in loss of tetrameric conformation and reduced enzymatic activity compared to that with rTET-NA^30^.

### rTET-NA immunogenicity in ferrets

We also evaluated the immunogenicity of rTET-NA in influenza naïve and pre-infected ferrets. In our study, naïve ferrets were vaccinated intramuscularly twice (21 days apart) with 5 µg or 45 µg of N2 rTET-NA derived from A/Singapore/INFIMH-16-0019/2016 or N1 rTET-NA derived from A/Michigan/45/2015 (500 µL/dose) with or without AF03 adjuvant. Alternatively, in the pre-immune model, ferrets were intranasally infected with influenza virus (1 mL/dose, split evenly between nostrils) before being vaccinated once as described with the homologous rTET-NA dose (0.36 to 45 µg) or with IIV (1.8 or 9 µg) and no adjuvant. NAI antibody titers were measured in sera three weeks after each intervention.

**Fig. 3:**
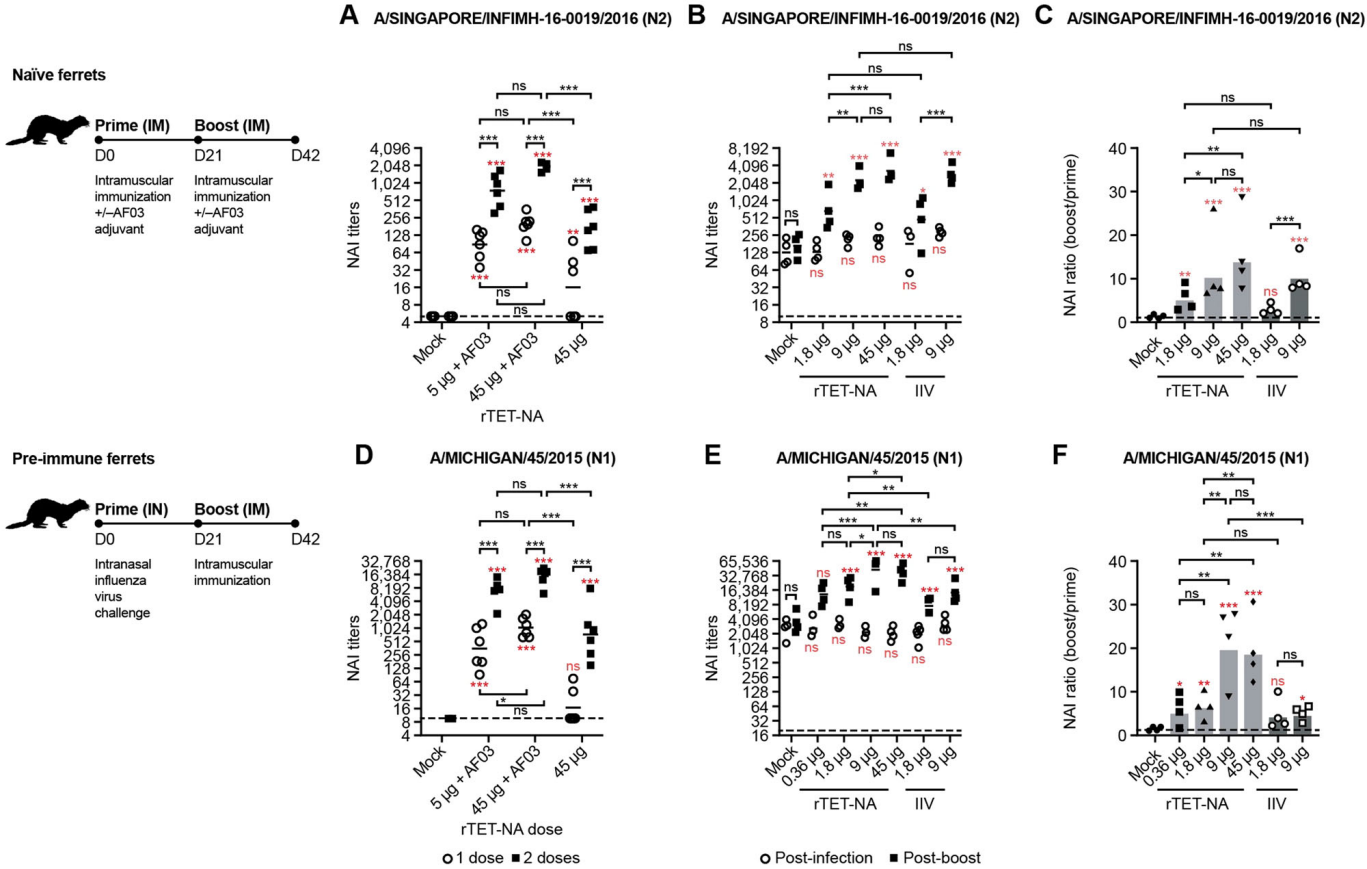
Immunogenicity of A/Singapore/INFIMH-16-0019/2016 N2 and A/Michigan/45/2015 N1 rTET-NAs in naïve and pre-infected ferrets. Sera were collected three weeks after initial dose or prime and three weeks after booster vaccination and tested via ELLA to assess NAI antibody activity using reassortant H6N2 A/Singapore/INFIMH-16-0019/2016 (**A**–**C**) or H6N1 A/Michigan/45/2015 (**D**–**F**) viruses as sources of sialidase, respectively. In **A**, **B** and **D**, **E**, º symbol represents IC_50_ NAI titers three weeks after initial prime (D21), ■ represents IC_50_ NAI titers three weeks after booster (D42), while dashed lines represent starting sera dilution or lower limit of detection for ELLA assay. The solid lines represent the average measurement. In **C** and **F**, all symbols represent boost/prime ratio for individual animals in the indicated vaccine groups, while the bar represents average ratio per vaccine group. *p < 0.05; **p < 0.01; ***p < 0.001 (the red text/symbols are comparisons vs PBS/control).

rTET-NA was highly immunogenic in naïve ferrets after a single dose and the responses were further boosted with a second dose (Fig. 3A, D). As with the mouse model, NAI titers were significantly enhanced with AF03 relative to the unadjuvanted formulation at the 45 μg dose. Furthermore, the lower dose of 5 μg with AF03 adjuvant elicited comparable NAI titers to the 45 μg with AF03 adjuvant after second dose, suggesting dose sparing potential by addition of adjuvant. Vaccination of pre-infected ferrets with a single dose of unadjuvanted A/Singapore/INFIMH-16-0019/2016 N2 rTET-NA or A/Michigan/45/2015 N1 rTET-NA resulted in comparable or superior post-boost NAI responses than a matched IIV dose (Fig. 3B, E). Similar to naïve mice, N1 rTET-NA showed significantly higher NAI titers than IIV in the primed ferret model at both doses tested (1.8 μg and 9 μg), demonstrating expanded benefit of the rNA platform for the N1 subtype (Fig. 3E). Boosting of NAI responses following infection (NAI ratio boost/prime) was also greater with N1 rTET-NA than IIV at the highest common dose assessed (9 μg; *P* < 0.001), but not at the lower dose assessed (1.8 μg) (Fig. 3F).

**Fig. 4:**
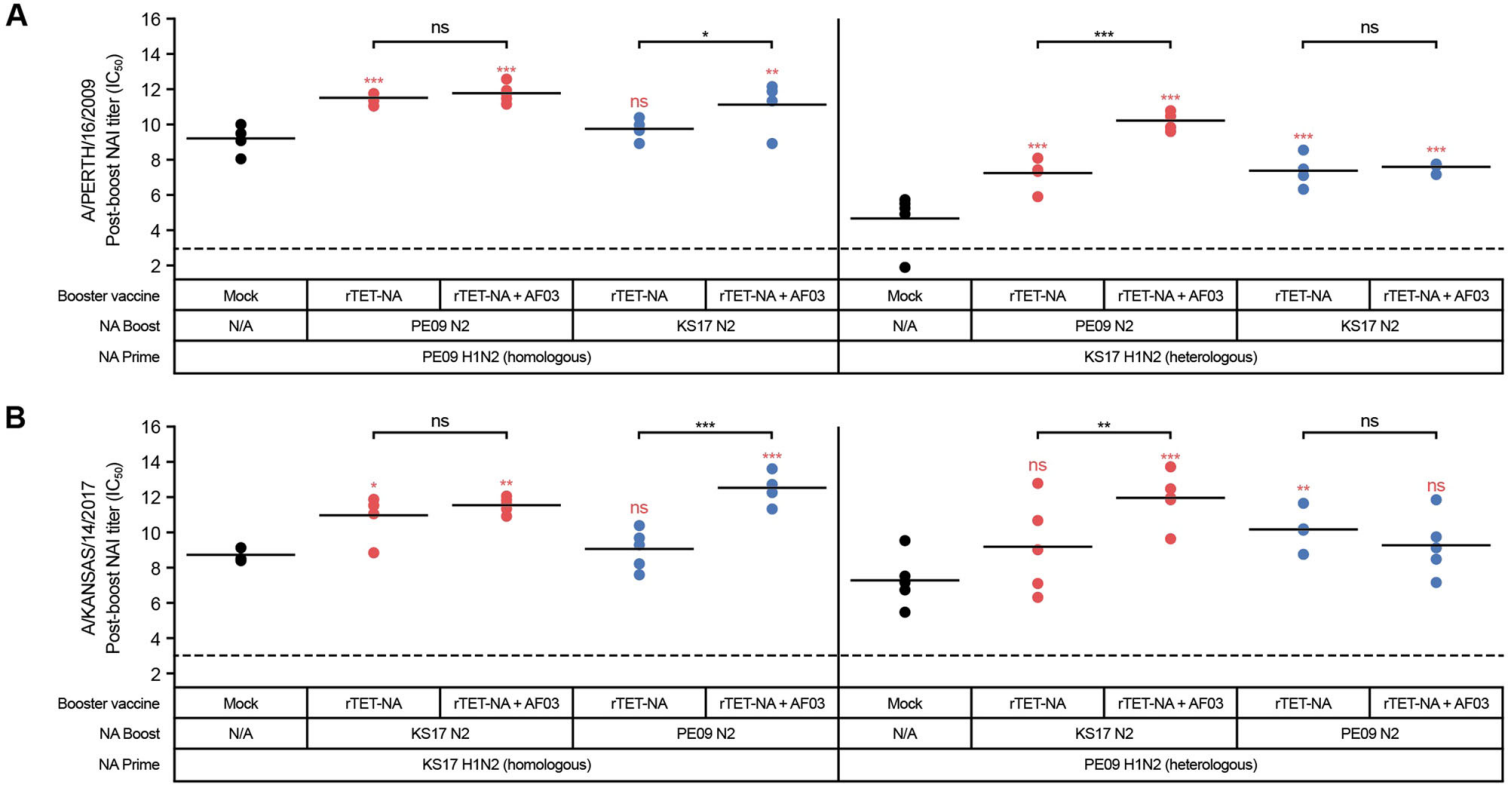
rTET-NA is highly immunogenic irrespective of the level of pre-immune mismatch, with adjuvant contributing to breadth of NAI responses. Ferrets were primed intranasally with A/Perth/16/2009 (PE09) or A/Kansas/14/2017 (KS17) H1N2 reassortant viruses, prior to receiving booster vaccination with PE09 rTET-NA or KS17 rTET-NA three weeks later. Sera were collected three weeks after booster vaccination and tested with ELLA to assess NAI antibody activity using A/Perth/16/2009 (**A**) or A/Kansas/14/2017 (**B**) H6N2 reassortant viruses as sources of sialidase, respectively. In (**A**, **B**), circles represent individual IC_50_ NAI titers three weeks after booster vaccination and solid lines represent average NAI titers per vaccine group, while dashed lines represent starting serum dilution or lower limit of detection for ELLA assay. *p < 0.05; **p < 0.01; ***p < 0.001 (the red text/symbols are comparisons vs PBS/control).

Vaccination of pre-infected ferrets with a single dose of adjuvanted A/Kansas/14/2017 N2 rTET-NA or A/Perth/16/2009 N2 rTET-NA boosted both homologous and heterologous NAI responses, irrespective of pre-existing immunity (Fig. 4). AF03 adjuvant significantly increased the NAI boosting effect in pre-infected ferrets exposed to partially matched vaccine (e.g., ferrets infected with heterologous strain and then vaccinated with homologous rTET-NA, or ferrets infected with homologous strain and then vaccinated with heterologous rTET-NA), but not in ferrets pre-infected and then immunized with matching NA. These data suggest that rTET-NA may provide broad protection against heterologous influenza viruses and that AF03 adjuvant can help expand the breadth of NAI responses in the event of vaccine mismatch.

### rTET-NA protection against challenge

**Fig. 5:**
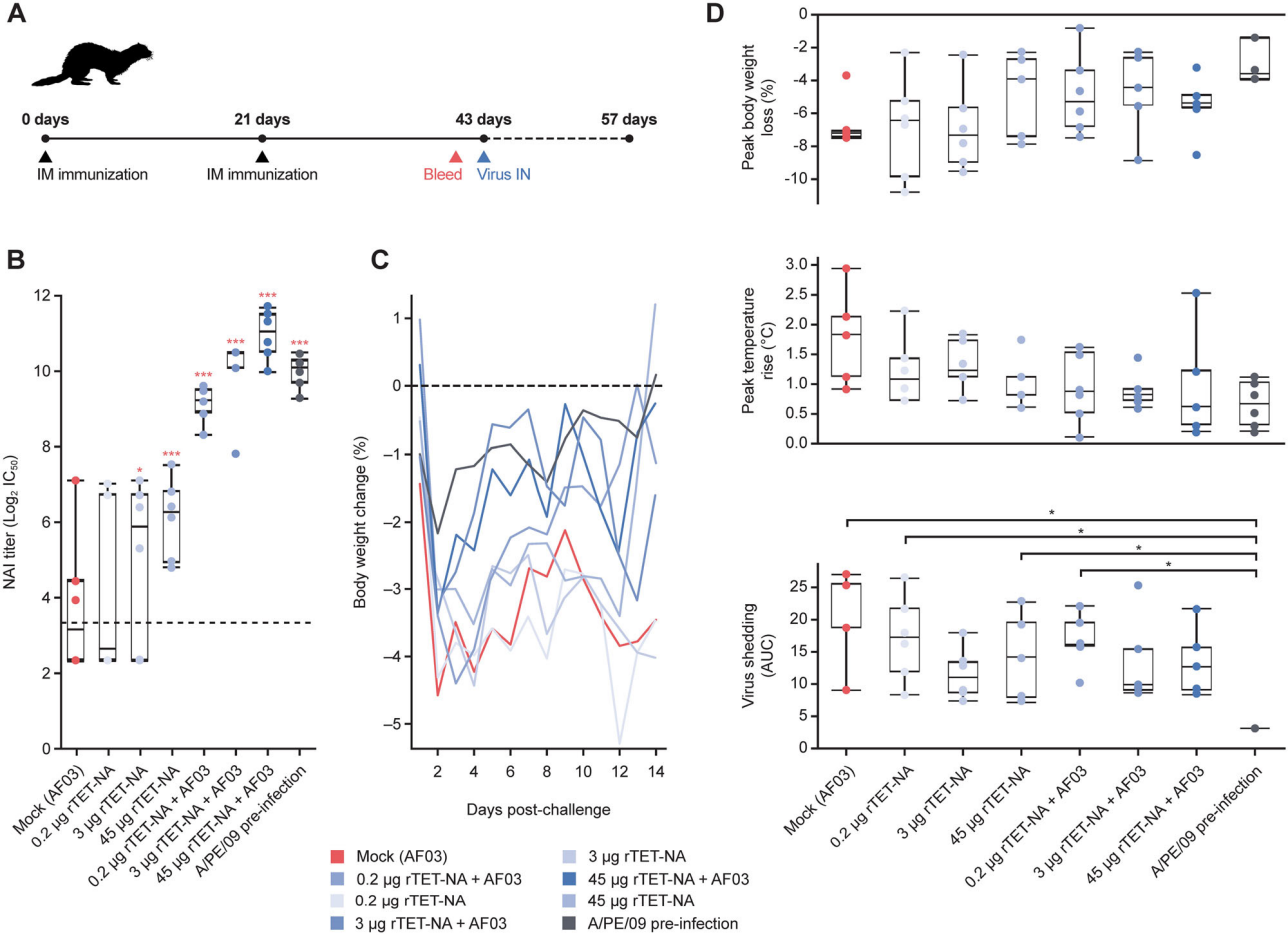
rTET-NA protects against challenge with H3N2 influenza virus. Naïve ferrets were vaccinated twice with A/Perth/16/2009 (PE09) rTET-NA (0.2–45 μg, +/− AF03), challenged intranasally with PE09 H3N2 wild-type influenza virus three weeks after last immunization and monitored for disease symptoms and viral shedding for additional two weeks (**A**). Pre-challenge PE09 anti-N2 NAI titers were measured in sera collected three weeks after second vaccination (**B**). Longitudinal analysis of mean body weight loss up to 14 days post-challenge (**C**). Cumulative body weight change (AUC), peak temperature rise and cumulative viral shedding were also determined for each vaccination group (**D**). Data shown for individual animals, with whiskers representing the upper and lower ranges, and the upper and lower box edges representing the upper and lower quartiles, respectively, and the median value shown by the lines splitting the box. Only significant differences shown: *p < 0.05; **p < 0.01; ***p < 0.001 (the red text/symbols are comparisons vs PBS/control).

We assessed the immunogenicity of two doses of rTET-NA three weeks apart in ferrets and subsequent protective effect to challenge with H3N2 influenza virus three weeks after the second dose (Fig. 5A). As previously shown for A/Singapore/INFIMH-16-0019/2016 (Fig. 3B), rTET-NA based on A/Perth/16/2009 N2 elicited dose-dependent NAI titers that were boosted by AF03 (Fig. 5B); both 3 and 45 μg rTET-NA adjuvanted doses induced NAI titers comparable to pre-infection with A/Perth/16/2009 virus at time of challenge, with no significant differences between these groups. All other rTET-NA doses (with or without AF03) induced significantly lower NAI titers than pre-infection only. rTET-NA vaccination protected against disease severity following challenge with wild-type influenza A/Perth/16/2009 H3N2 virus expressing homologous NA in ferrets by reducing intensity and duration of clinical signs such as body weight loss and fever (both not statistically significant) and overall viral shedding (Fig. 5C, D).

Longitudinal analysis of body weight (Fig. 5C) and temperature changes (not shown) showed that the majority of animals developed peak symptoms by day 2 post-challenge, independent of the study group. Animals vaccinated with adjuvanted vaccine seemed to recover body weight faster than those who received mock (adjuvant alone), suggesting that vaccination may reduce disease duration. However, differences between vaccinated/pre-infected and mock groups were not significant due to variability in late-onset or delayed symptoms experienced by some of the animals during the second week post-challenge. Although rTET-NA does not appear to be as efficient as prior infection against viral shedding (Fig. 5D), this result is expected since infection provides both anti-NA and anti-HA immunity in addition to T-cell immunity against conserved epitopes^31^. Interestingly, NAI based on prophylaxis or treatment with oseltamivir consistently improves disease symptoms both in humans and ferrets, while exhibiting varying effect on virus shedding ranging from complete prevention of infection in the case of prophylactic administration to partial reduction in virus shedding when administered therapeutically^32,33^, similar to our observation with rTET-NA vaccination.

**Fig. 6:**
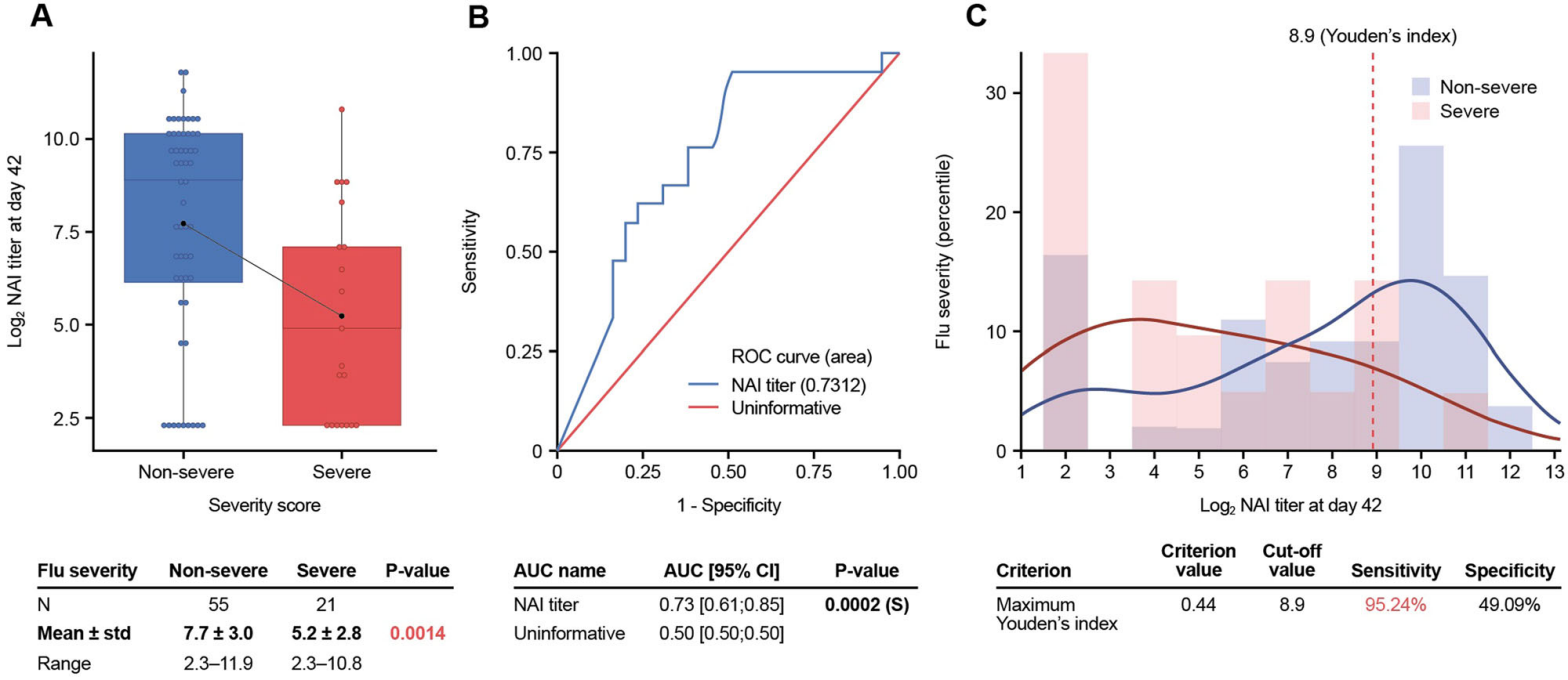
Post-vaccination NAI titers are a correlate of protection in the ferret model. **A** Ferrets with severe symptoms showed significantly lower NAI titers than those classified as non-severe. **B** NAI titers can correctly predict disease severity in ferrets since the area under the curve (AUC) of the receiver operating characteristics (ROC) curve for NAI titers was significantly higher than an uninformative biomarker. **C** Log2 NAI titer of 8.9 (NAI titer of 477) was the optimum NAI titer cutoff at D42 that best distinguishes severe from non-severe cases as determined by the Youden index.

Post-vaccination NAI titers were inversely correlated with disease severity and had predictive power to be used as a correlate of protection (Fig. 6). The association between NAI titers and disease severity was examined by developing a disease scoring system based on the distribution of peak symptoms and overall viral shedding (Table S1). Ferrets were classified as having severe or non-severe disease, based on their combined symptom severity score, with the distribution of NAI titers analyzed according to this binary disease classification (Fig. 6A, C). Ferrets with severe symptoms showed significantly lower NAI titers than those classified as non-severe, although some ferrets with titers below the limit of detection were also protected (Fig. 6A). To further assess NAI as a biomarker in the prediction of disease severity, receiver operating characteristics (ROC) analysis was undertaken. The area under the curve (AUC) of the ROC curve for NAI titers was significantly higher than an uninformative biomarker, providing evidence that NAI titers could be used to correctly predict disease severity in ferrets (Fig. 6B). The optimum NAI titer cutoff at D42 that best distinguishes severe from non-severe cases determined by the Youden index was a Log2 NAI titer of 8.9 (NAI titer of 477) (Fig. 6C). Thus, a ferret with severe symptoms has a probability of having a Log2 NAI titer value ≥ 8.9 (i.e. NAI titer ≥478) estimated at 95% (sensitivity), while a ferret without severe symptoms has a probability of having a Log2 NAI titer value < 8.9 (i.e. NAI titer <478) estimated at 49% (specificity).

### Impact of rTET-NA vaccine supplementation on HA and NA responses

**Fig. 7:**
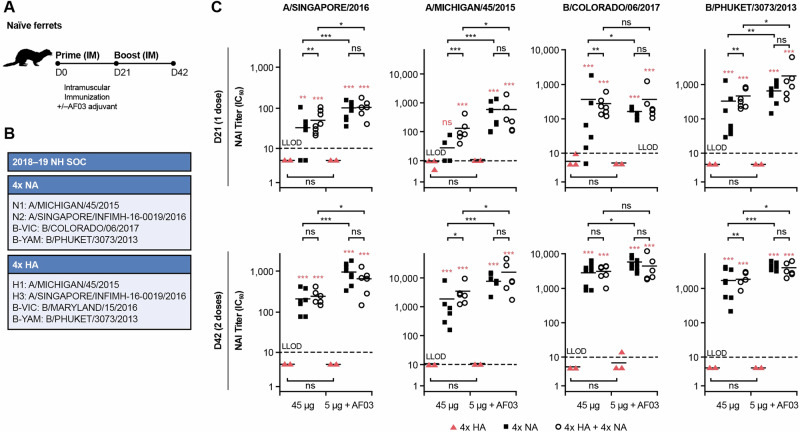
rTET-NA retains its immunogenicity following octavalent HA and NA vaccination in ferrets. Naïve ferrets were vaccinated twice (21 days apart; (**A**)) with quadrivalent rTET-NA plus quadrivalent baculovirus-HA (**B**) from strains included in the quadrivalent 2018–19 Northern hemisphere seasonal influenza vaccine. NAI titers against vaccinal N2, N1, B/Victoria/2/87-like and B/Yamagata/16/88-like strains were determined in sera from vaccinated ferrets three weeks after each immunization (**C**). *p < 0.05; **p < 0.01; ***p < 0.001 (the red text/symbols are comparisons between 4xHA and 4x NA or 4xHA + 4xNA at matched dose and time). For all comparisons between 1 dose and 2 doses, all groups that received NA-containing formulations had significantly higher NAI titers after 2 doses (*p* < 0.001), but there were no significant differences for groups that received formulations containing only HA (4xHA). The solid lines represent the average measurement, and the dashed lines indicate the lower limit of detection (LLOD).

We showed that rTET-NA retains its immunogenicity following octavalent HA and NA vaccination in ferrets; this is the first demonstration of the potential of a fully recombinant octavalent vaccine containing both HA and NA. Ferrets immunized with quadrivalent rTET-NA plus quadrivalent baculovirus-HA developed NAI antibody responses of similar magnitude to animals immunized with quadrivalent rTET-NA mixture alone, in a dose- and adjuvant-dependent manner (Fig. 7). No interference was observed between HA and NA antigens, although some synergistic effects were detected for the 45 µg dose with no adjuvant after the first vaccination. No NAI titers were detected in the groups that received HA-only formulations. Significant NAI titer increases were observed after the second vaccination. The induction of NAI and NA ELISA titers (Fig. S2) was confirmed across all NA subtypes assessed, including from the two influenza B virus lineages.

**Fig. 8:**
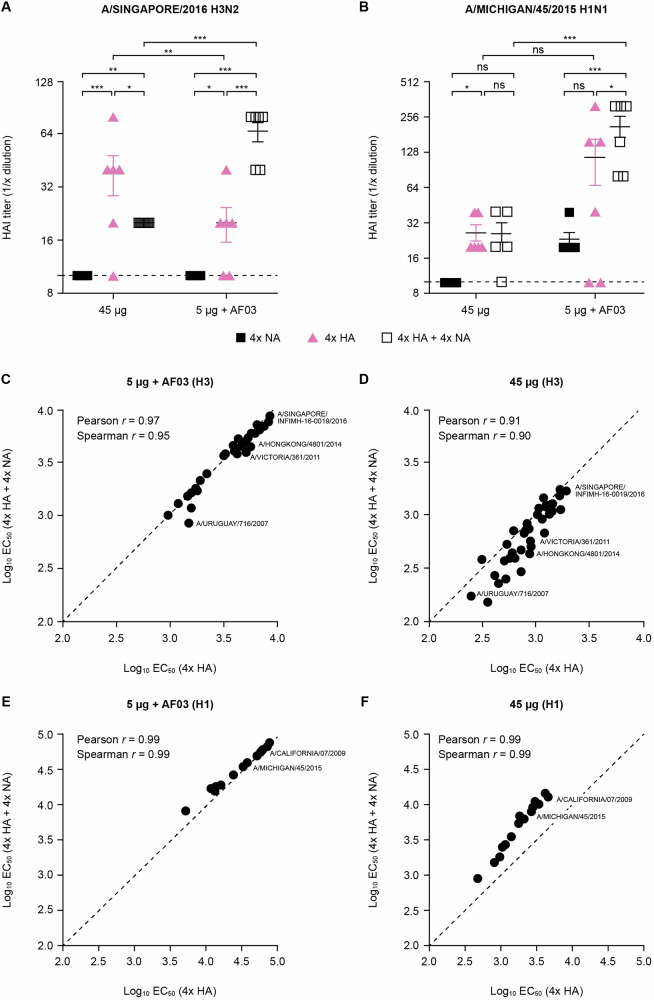
rTET-NA supplementation does not interfere with the magnitude and breadth of HA responses elicited by octavalent recombinant HA and NA vaccine. HAI titers against vaccinal H1 and H3 strains (**A**, **B**) as well as total anti-HA antibody titers against panel of seasonal H3 (**C**, **D**) and H1 (**E**, **F**) strains were determined in sera from vaccinated ferrets three weeks after the second immunization. The line and whiskers represent the mean and 95% CI. *p < 0.05; **p < 0.01; ***p < 0.001 (the red text/symbols are comparisons vs 4x NA group at matched dose and time).

HA-specific antibody responses in octavalent vaccinated ferrets were generally similar (unadjuvanted 45 μg; for A/Michigan/45/2015 H1 only) or superior (5 μg with AF03 adjuvant; for both A/Michigan/45/2015 H1 and A/Singapore/2016 H3) to ferrets immunized with quadrivalent baculovirus-HA vaccine alone, both in terms of quantity and quality as demonstrated by HAI and multiplex antibody-binding ELISA (antibody forensics, AF) (Fig. 8). Similar results to those for NAI described above were demonstrated for HAI against vaccinal strains: lack of interference between HA and NA antigens, and a dose-sparing effect of AF03. A potential synergistic effect at the 5 μg + AF03 dose was also observed (Fig. 8A, B). Multiplexed ELISA analysis confirmed the breadth of HA antigens was preserved upon addition of NA and showed that AF03 increased the magnitude and breadth of total anti-HA antibody responses for both HA-only and NA-supplemented HA vaccines (Fig. 8C–F).

## Discussion

In this study, we developed rTET-NA (N1 or N2 subtype) proteins for multiple influenza subtypes and demonstrated that vaccination with these antigens can reliably induce robust anti-NA immunity, at least similar to other viral NA-containing preparations, in both naïve mice and ferrets. In both models, inclusion of AF03 adjuvant greatly boosted anti-NA immunity induced by rTET-NA and virus-derived NA. rTET-NA was also highly immunogenic in ferrets with pre-existing immunity against influenza, irrespective of sequence homology between the virus prime and rTET-NA vaccine boost, and provided protection against influenza-like disease symptoms. Assessing rTET-NA immunogenicity in ferrets with pre-existing immunity against influenza was done to replicate the situation with vaccination in humans who predominantly have some pre-existing anti-influenza immune memory due to earlier infections with homologous subtypes and/or vaccination. Of note, post-vaccination NAI titers were inversely correlated with disease severity and had predictive power to be used as a correlate of protection in the context of influenza pre-immune ferrets. The addition of rTET-NA to a HA-based influenza composite vaccines induced robust NA-specific humoral immunity in ferrets while retaining the ability to induce HA-specific immunity.

We and others have previously observed that functional tetrameric rTET-NA was important for inducing effective NA immunity^18,34,35^. Monomeric NA based on rNA lacking the tetramerization domain or rNA purified from monomeric fractions had reduced immunogenicity in naïve animals (data not shown), consistent with previous observations that the NA tetramer is more immunogenic and provides better protection than the monomer^18,34^. Similarly, others have shown that the immunogenicity of NA in IIVs correlates with NA enzymatic activity (as a proxy of correct folding), though the magnitude of responses was strain-specific^36^. However, it has also been shown that lack of NA activity in correctly folded protein does not lead to loss of immunogenicity^18,35^. In addition, monoclonal antibodies against highly conserved conformational NA epitopes (i.e. NAI) appear protective against influenza challenge^37^.

The benefits of rNA technology include more reliable recovery of functional tetrameric NA across all subtypes (and lineages), and better-defined doses than split-inactivated influenza vaccines. The inactivation process with the latter may impact on critical conformational NA epitopes and further processing may lead to variability in the content of NA and other viral proteins^38^. Thus, the use of rNA may lead to greater consistency in vaccine immunogenicity and safety. Importantly, our rTET-NA constructs were produced as tetramers, with correctly folded and enzymatically active proteins, for all four circulating NA influenza virus strains, as needed for seasonal vaccination.

We observed that AF03 further increased antibody responses to rTET-NA in both mice and ferrets. Of note, AF03 was rTET-NA dose-sparing in our animal models, and is consistent with dose-sparing effects reported with pandemic influenza A vaccines in humans^39,40^. The mechanism underlying adjuvant activity remains unknown, but is thought to be due to emulsion vehicle properties and recruitment of antigen-presenting cells^39^.

Our results suggest that NA-only vaccines should reduce disease severity following challenge with an NA-matched influenza virus strains, even in the absence of HA immunity. This result suggests that NA addition to existing HA vaccines may increase vaccine efficacy even in the event of strain mismatch. We also demonstrated that post-vaccination NAI titers had predictive power to be used as a biomarker of disease severity. This observation is consistent with other studies in both ferrets^41^ and humans^12–14^ that showed NAI titers were strong correlates of protection^12–14,41^, and thus further supports the use of ferrets as a proof of concept influenza translational model. Of note, NAI titers appear to be a more robust correlate of protection than HAI titers in human influenza^12,13^. However, utilizing both NAI and HAI titers may be better for ascertaining protection than either alone, especially in the elderly^14^.

The degree of homology between the vaccine NA and challenge influenza virus may be an important determinant of protection^12,41^. While our study shows that rTET-NA protects against homologous influenza challenge, NA-based immunity was previously shown to provide partial protection from challenge with heterologous but not from heterosubtypic influenza A^42^. In our study, some ferrets were protected even in the absence of detectable homologous NAI titers (Fig. 6C) or NA ELISA titers (Fig. S3). This is consistent with earlier studies that established baseline HAI as a correlate of protection^43^, suggesting that other protective factors may also be involved. For instance, heterosubtypic cross-protection in ferret studies correlated with cross-reactive interferon-gamma-secreting lymphocytes^44^, while innate and adaptive cellular responses were also shown to correlate with protection against symptomatic influenza disease in humans^15^.

Immune responses to NA have been shown to be blunted in mice following immunization with conventional IIV or live-attenuated influenza vaccine (LAIV)^45^. This has been attributed to intravironic competition, where the immune response to the more abundant HA outcompetes NA antibody responses (i.e., HA dominant immunity), thus losing protection afforded by the latter. However, in mice, this HA dominant immunity can be overcome with comparable HA and NA amounts, resulting in equivalent immunogenicity and homotypic protection, as well as broader heterovariant protection than conventional IIV or LAIV^45,46^. In ferrets, we showed that rTET-NA was immunogenic irrespective of pre-existing HA immunity or vaccine mismatch. In addition, consistent with other studies^45,46^, we confirmed that rTET-NA retains its immunogenicity when HA and NA are co-administered in equivalent amounts. Similarly, we demonstrated that rTET-NA supplementation does not interfere with the magnitude and breadth of HA immunogenicity in ferrets. We also showed that HA- and NA-specific antibody responses induced by a fully recombinant octavalent vaccine targeting both HA and NA antigens to all four circulating influenza virus strains, as needed for a seasonal vaccine, were similar to those induced by the respective quadrivalent rHA or rNA antigen only vaccines.

In conclusion, our results demonstrate that the addition of recombinant NA components (a rTET-NA design for proof of concept in this study) is a viable option to increase the immunogenicity and potential efficacy of already licensed influenza vaccines by means of boosting NA immunity.

## Methods

### Ethics

Animal experiments were carried out in compliance with the Public Health Service Policy on Humane Care and Use of Laboratory Animals^47^ and the Guide for the Care and Use of Laboratory Animals^48^, and were conducted with approved animal protocols from the Sanofi Institutional Animal Care and Use Committee. All animals were housed under specified pathogen-free conditions with food and water *ad libitum*, and acclimatized for 3 days (mice) or 7 days (ferrets) before entering the studies. Any ferret judged to be moribund, and where euthanasia was warranted as judged by a trained veterinarian (or undertaken as part of the study), were anesthetized and administered an overdose of euthanasia agent containing pentobarbital or other agents approved by American Veterinary Medical Association for euthanasia.

### Influenza viruses

Reassortant H6 viruses used in the enzyme-linked lectin assay (ELLA) were generated by reverse genetics, with each reassortant expressing the targeted NA antigen, the HA from A/mallard/Sweden/81/2002 H6N1, and internal genes from A/Puerto Rico/8/1934 H1N1 [PR8]. Reassortant H1 viruses used in pre-infected ferret models were also generated by reverse genetics, with each reassortant expressing the targeted NA antigen, and the HA and internal genes from A/Puerto Rico/8/1934 H1N1 [PR8]. HA and NA segments including non-coding regions were generated by custom gene synthesis (Geneart AG), and PR8 segments were derived from a viral isolate as previously published^49^. All segments were cloned into a bi-directional transcription plasmid derived from pUC57 (Genscript) through the incorporation of polymerase (Pol) I and Pol II promoters, as described elsewhere^49^. Briefly, 293FT cells (Thermo Fisher Scientific) were transfected with a total of eight plasmids representing each influenza virus segment using Lipofectamine 2000 CD (Thermo Fisher Scientific). After 24 h, Madin-Darby canine kidney Cells (MDCK-) adult T-cell leukemia (ATL) cells (ATCC) were added to the transfected cells in the presence of tosyl phenylalanyl chloromethyl ketone (TPCK)-treated trypsin (Sigma) to allow influenza virus propagation. Cell culture supernatants containing influenza virus were harvested 7 days post-MDCK addition and passaged in 8–10-day-old embryonated chicken eggs (Charles River Laboratories, Inc.). Inoculated eggs were incubated at 37 °C for 48 h, then cooled to 4 °C for 12 h, harvested, and clarified by low-speed centrifugation (3000 rpm, 20 min). Virus titers were determined by plaque assay on MDCK cells.

Egg-grown stocks of A/Michigan/45/2015 (H1N1), A/Singapore/INFIMH-16-0019/2016 (H3N2), B/Colorado/06/2017 (B/Victoria/2/87-like lineage) and B/Phuket/3073/2013 (B/Yamagata/16/88-like lineage) included in HAI testing were provided by Sanofi Global Clinical Immunology (Swiftwater, PA). Wild-type influenza A/Perth/16/2009 (H3N2) used in the ferret challenge study was provided by IIT Research Institute (Chicago, IL). All viruses were stored at <–65 ^o^C until use.

### Vaccine antigens

Constructs were designed for the expression of recombinant, soluble influenza NA. Both tetrameric and monomeric NA construct design included an N-terminal CD5 secretion signal peptide, 6HIS tag and the globular neuraminidase head domain similar to other rNA constructs described elsewhere^29^. The tetrameric design also contains a tetrabrachion domain between the HIS tag and the globular head for multimerization. Using a defined amino acid sequence, a codon optimized synthetic gene was assembled from oligonucleotides and/or PCR products and the fragment was inserted into pcDNA3.4-TOPO (ThermoFisher). The plasmid DNA was purified from transformed bacteria and scaled to achieve appropriate concentration for transfection. Protein expression was performed in CHO-S cells using the ExpiCHO™ Expression System Max Titer Protocol (ThermoFisher). A clarification step was performed to separate secreted proteins from cells. NA protein was purified from host-cell proteins by affinity (HisTrap HP Column – GE Healthcare) followed by anion exchange chromatography (HiTrap Q HP – GE HealthCare), dialysis into 10 mM phosphate buffered saline (pH 7.2) and sterile filtration through a 0.2 μm filter. The NA vaccine preparations were produced in compliance with the current good research practices (cGRP).

Recombinant HA proteins were obtained from Protein Sciences. Briefly, purified HA proteins were produced by baculovirus expression in a continuous insect cell line (EXPRESSF + ^®^) derived from Sf9 cells and grown in serum-free medium. IIV was prepared from influenza virus propagated in embryonated chicken eggs, which was subsequently inactivated using formaldehyde, then concentrated, and purified by zonal centrifugation on a sucrose gradient, split with Triton^®^ X-100, before repeat purification and resuspension in isotonic sodium phosphate-buffered sodium chloride solution. Preparations were sterile filtered using a 0.2 μm syringe filter. Live influenza virus-derived neuraminidase (LVNA) was isolated from influenza virus propagated in embryonated chicken eggs. Virus was purified by sucrose gradient ultracentrifugation and NA was extracted by detergent solubilization, further purified by column chromatography, and suspended in sodium phosphate-buffered isotonic sodium chloride solution. Preparations were sterile filtered using a 0.2 µm syringe filter.

### Size exclusion chromatography with multiangle light scattering (SEC-MALS): assessment of molecular weight and purity of secreted/soluble NA

Size exclusion chromatography was carried out on a Waters ACQUITY Arc Bio HPLC system with a TSK-GEL G4000 PWXL (7.8 mm × 30 cm) column (Tosoh Bioscience) using phosphate buffer saline containing 0.02% sodium azide (pH 7.4) as mobile phase. A flow rate of 0.5 mL/min was used. The effluent was detected by a UV detector at 280 nm, followed by a Wyatt DAWN MALS detector (HELEOS II) and an Optilab TrEX differential refractive index (RI) detector. Empower^®^ (Waters Corporation, Milford, MA) software was used for high performance liquid chromatography (HPLC) control and data analysis on the UV chromatogram. The purity of the sample was calculated based on the percentage of the specific peak area/the total peak areas. ASTRA software was used for MALS data collection and protein molecular weight determination using light scattering signals with a concentration detector (RI or UV).

### 2′-(4-methylumbelliferyl)-α-D-N-acetylneuraminic acid (MUNANA)-based assay: assessment of NA activity

The MUNANA-based assay was based on a previously described method with modifications^50^. Briefly, two-fold serial rTET-NA dilutions were prepared in 96-well plates using buffer (33.3 mM 2-[N-morpholino] ethanesulfonic acid [MES, pH 6.5], 4 mM CaCl2, 50 mM BSA) and mixed with MUNANA substrate (100 µM) and incubated for 1 h at 37 °C with shaking. The reaction was stopped by addition of alkaline solution (0.2 M Na_2_CO_3_). The fluorescence intensity (RFU, relative fluorescence unit) from the rTET-NA and MUNANA substrate mixture was measured using excitation and emission wavelengths of 355 nm and 460 nm, respectively. A standard curve was generated using 4-methylumbelliferone (4-MU) diluted in enzyme buffer at various concentrations; rTET-NA enzymatic activity was determined against a 4-MU reference with the results expressed in µM/60 min for total NA activity and nmol/min/µg for specific NA activity.

### Oseltamivir-binding assay: detection of tetrameric NA

The oseltamivir phosphate (Tamiflu)-NA binding assay was performed on Octet-Red96 instrument (ForteBio, Sartorius), an Octet BLI Detection System using the bio-layer interferometry (BLI) technique that facilitates real-time label-free analysis for the determination of kinetics, affinity, and quantitation regarding the NA bound to the streptavidin biosensor tip coated with oseltamivir-phosphate–biotin conjugate. In brief, oseltamivir-phosphate–biotin conjugate (5–10 μg/ml) in 1xKB buffer (containing PBS pH 7.4; 0.02% Tween-20; 0.1% albumin; and 0.05% sodium azide) is first captured on a streptavidin-coated biosensor. The interaction between NA and oseltamivir-phosphate was then initiated by dipping oseltamivir-phosphate bound biosensors into sample wells containing a 2-fold dilution series of recombinant NA (0.16–40 μg/ml in 1xKB buffer). The binding between oseltamivir-phosphate and NA produces a measured shift in the interference pattern via the detector. The level of binding response is proportional to the concentration of NA. An Octet data acquisition software was used for instrument control and data collection (ForteBio, Sartorius), and an Octet Data Analysis software was used for data process (ForteBio, Sartorius).

### Mouse studies

Female BALB/c mice (8 per group; sample size chosen to ensure 85% power of detecting a difference between groups) aged 6–8 weeks and with a body weight of 15–20 g (Charles River) were vaccinated twice 21 days apart with the same dose (0.2 or 1 μg) of various NA-containing preparations (50 µL/dose, intramuscularly) in the presence or absence of AF03 adjuvant containing 5% squalene (Sanofi). The mice were not anesthetized before any in-life study procedure. Terminal bleed (via cardiac puncture) was undertaken after isoflurane overdose exposure followed by cervical dislocation two weeks after booster vaccination; sera pools from two animals were created (stored at –20 °C until required), resulting in a total of 4 samples per group. The sera were tested by ELLA to assess NAI activity or via ELISA to derive NA-binding or tetrabrachion-binding antibodies.

### Ferret studies

#### Immunization studies

Outbred male and female Fitch ferrets (Marshalls Farms North Rose, NY), aged 17–21 weeks old, with a bodyweight of at least 600 g, and seronegative (by HAI antibody assessment) to the four seasonal influenza vaccine strains, were used for vaccination studies. The ferrets were randomized into study groups (6 per group) using a body weight stratification procedure that produced groups with similar mean body weights. For standard immunogenicity assessments, naïve ferrets were vaccinated twice 21 days apart with the same dose (5 µg or 45 µg) of various NA-containing preparations (500 µL/dose, intramuscularly) with or without AF03 adjuvant. Animals used to assess impact of pre-existing influenza immunity on NA immunogenicity were initially primed by intranasal challenge with reassortant influenza virus A/Perth/16/2009 H1N2 or A/Kansas/14/2017 H1N2 on day 0 (1000 µL/dose, split evenly between nostrils). Three weeks after initial infection, animals received a single dose (1.8 to 45 µg) of A/Perth/16/2009 rTET-NA or A/Kansas/14/2017 rTET-NA (500 µL/dose, intramuscularly) with or without AF03 adjuvant.

The animals used to assess multivalent vaccine immunogenicity were vaccinated twice 21 days apart with a mixture of four rTET-NA antigens, each antigen comprising the NA head domain from one of the strains included in the quadrivalent 2018-19 seasonal influenza vaccine (A/Singapore/INFIMH-16-0019/2016 (N2), A/Michigan/45/2015 (N1), B/Colorado/06/2017 (B/Victoria/2/87-like lineage), and B/Phuket/3073/2013 (B/Yamagata/16/88-like lineage)). All antigens were administered at a 1:1 ratio, without adjuvant (45 µg/antigen dose) or adjuvanted with AF03 (5 µg/antigen and 45 µg/antigen doses).

All ferrets were bled under sedation at baseline and three weeks after primer (one day before or just before booster) and booster vaccination, and two weeks after challenge as required. For all in-life procedures, the ferrets were anesthetized via intramuscular (IM) injection with a ketamine HCl (25 mg/kg) and dexmedetomidine (0.001 mg/kg) solution. For euthanasia, ferrets were sedated with the same anesthetic cocktail as above, blood was collected, and then animals were administered an overdose of euthanasia agent containing pentobarbital (e.g., Beuthanasia-D) or other American Veterinary Medical Association approved method of euthanasia. Sera samples (stored at –20^o^C until required) were tested by ELLA to assess NAI activity. Additionally, the HAI assay and antibody forensics were undertaken to assess antibody responses to hemagglutinin antigens following multivalent vaccination.

#### Challenge studies

Outbred male Fitch ferrets aged 17–21 weeks old, with a bodyweight of at least 600 g, and seronegative (by HAI antibody assessment) to the four seasonal influenza vaccine strains (Triple F farms, Sayre, PA), were randomized into study groups (16 per group) using a body weight stratification procedure that produced groups with similar mean body weights. For all in-life procedures, the ferrets were anesthetized via IM injection with a ketamine HCl (25 mg/kg) and xylazine (2 mg/kg) mixture. Ferrets were initially vaccinated twice 21 days apart with the same dose (0.2 µg to 45 µg) of A/Perth/16/2009 N2 rTET-NA (500 µL/dose, intramuscularly) with or without AF03 adjuvant. Three weeks after booster vaccination, the ferrets received intranasal challenge with 10^7^ PFU of A/Perth/16/2009 H3N2 wild-type influenza A virus (1000 µL/dose, split evenly between nostrils). Prior to intranasal administration, ferrets were anesthetized with a ketamine HCl (25 mg/kg) and xylazine (2 mg/kg) mixture. The ferrets were then held upright and 0.5 mL of the inoculum was administered dropwise into each nostril (for a total of 1.0 mL/ferret). The animals were monitored for 14 days post-challenge for clinical symptoms and changes in body weight once daily, and body temperature twice daily. Nasal washes were collected from all challenged animals on days 1, 3, 5 and 7 post-challenge and samples were stored at ≤ –65 °C for viral shedding assessment. Selected ferrets (1–2) from each group were euthanized with an intravenous dose of Beuthanasia-D (150 mg/kg) on days 1, 3, 6 and 14 post-challenge and necropsied. Lungs and nasal turbinates from necropsied ferrets were collected for viral titer analyses.

### Viral titration of nasal wash and respiratory tissues

Nasal wash specimens were collected from experimentally infected ferrets on alternate days following intranasal challenge. Briefly, ferrets were initially anesthetized followed by 0.5 mL intranasal instillation into each nostril with sterile PBS containing gentamicin (50 μg/mL), penicillin (100 U/mL), and streptomycin (100 μg/mL), and collection of the nasal wash, which was then stored at ≤ –65 °C until assessment. Virus in the nasal wash specimens was titrated by standard 50% tissue culture infectious dose (TCID_50_) assay as follows. The nasal washes were thawed and then clarified by centrifugation. The resulting supernatant was 10-fold serially diluted and transferred to a 96-well plate for titration on a monolayer of MDCK cells. Sections of lungs (right and left cranial, and right left caudal lung lobes) and nasal turbinates harvested for viral titer assessment were weighed and flash frozen in an ethanol/dry ice bath or liquid nitrogen and stored at ≤ –65 °C until processing for virus titration by standard TCID_50_ assay as described.

### Enzyme-linked lectin assay (ELLA): assessment of NAI responses

NAI antibody responses were measured against H6 reassortant viruses containing NA derived from strains of interest by ELLA as previously described^51^. Briefly, a H6 reassortant virus containing the NA derived from a strain of interest was titrated in fetuin-coated 96-well plates to determine the standard amount of virus that provides 70% of maximum NA enzymatic activity. Titration of NAI antibodies present in the sera was achieved by performing two-fold serial dilutions of heat inactivated sera. A total of 50 μL of diluted sera was then added to 50 μL of diluted virus corresponding to 70% of maximum NA enzymatic activity in a fetuin-coated plate. The serum-virus mixture was incubated at 37 ^o^C overnight. The plate was washed four times, incubated with horseradish peroxidase- (HRP-) conjugated peanut agglutinin (Sigma) and washed again prior to developing by addition of o-phenylenediamine dihydrochlorid. Low or no signal relative to a virus control indicates inhibition of NA activity due to the presence of NA-specific antibodies. NAI titers were approximated with a non-linear four parameter logistic (4PL) curve using GraphPad Prism software and the 50% maximal inhibitory concentration (IC_50_) calculated.

### Enzyme-linked immunosorbent assay (ELISA): assessment of NA-binding and tetrabrachion-binding titers

The rTET-NA derived from influenza virus strains A or B were immobilized on the surface of nickel coated 96-well microtiter plates (Pierce) by adding 50 μL of rTET-NA at working dilution of 0.5 μg/mL and coating overnight at 4^o^C. Coated plates were washed four times to remove any unbound protein and incubated with 5% w/v Blot-Quick Blocker (Biosciences) (300 μL per well) at room temperature for two hours to saturate the non-specific binding sites. After blocking, the plates were incubated with 50 μL of three-fold serially diluted individual serum samples at room temperature for two hours to allow binding of anti-NA antibody to immobilized rTET-NA protein. The plates were washed four times to remove any unbound antibody and incubated with 50 μL of HRP-conjugated anti-ferret or anti-mouse detection antibody (Abcam) and developed through incubation with 50 μL of the HRP enzyme substrate mix containing 3,3’,5,5’-tetramethylbenzidine and hydrogen peroxide. The reactions were stopped by addition of 50 μL of 0.16 M sulfuric acid (Thermofisher) and the absorbance at 450 nm read immediately. Titers were determined based on the serum dilution that achieves 50% binding (EC_50_) using a 4-parameter non-linear regression analysis from GraphPad Prism software.

For the assessment of antibodies that specifically bind to the tetrabrachion domain present in the r-TET-NA vaccines included in this study, recombinant protein containing a fragment of human serum albumin (domain III) fused to the tetrabrachion, and his-tagged at its N-terminus were immobilized on the surface of nickel coated 96-well microtiter plates and the assay undertaken in the same manner as described above.

### Hemagglutination-inhibition (HAI) assay

Sera were treated with receptor-destroying enzyme (RDE; Denka Seiken, Co., Japan) to inactivate nonspecific inhibitors prior to the HAI assay. Briefly, three parts RDE were added to one part serum and incubated at 37 °C overnight. RDE was subsequently inactivated by incubation with 6 times excess serum volume at 56 °C for 30 min, followed by 2-fold serially dilution in 0.9% saline in 96-well v-bottom microtiter plates. Equal volumes of each virus from the panel, adjusted to 4 hemagglutinating units per 250 μL, were added to each well. For the current study, homologous virus panel included A/Michigan/45/2015 (H1N1), A/Singapore/INFIMH-16-0019/2016 (H3N2), B/Colorado/06/2017 (B/Victoria/2/87-like lineage) and B/Phuket/3073/2013 (B/Yamagata/16/88-like lineage) viruses grown in eggs. The plates were covered, incubated for 20 min at room temperature, and 1% turkey red blood cells (Lampire Biologicals) in PBS subsequently added. The plates were agitated to mix the red blood cells, covered and allowed to settle at room temperature for 1 h. The reciprocal dilution attained for the last well containing non-agglutinated red blood cells was taken as the HAI titer.

### Antibody forensics assay

Antibody forensics methods were used to measure strain-specific rHA antibodies in ferret sera using magnetic bead array (MagPlex^®^ Microspheres) with fluorescent dyes (as described in Ustyugova et al.)^52^. The strength of antibody binding to strain-specific rHA was presented in normalized mean fluorescent intensity units, calculated from raw fluorescent intensity signal multiplied by the serum dilution. The rHAs coupled to the magnetic beads were selected based on antigenicity data published in the annual and interim reports on the composition of influenza vaccines^52^. In addition to 2018–2019 Northern hemisphere recommended strains, the rH3 panel included strains for 2013 through 2016 seasons, while the H1 panel encompassed strains from 2009 through 2016 seasons. A complete list of rHAs included in this study is provided in the supplementary materials. Individual ferret sera were analyzed and the resultant antibody forensics data for 40 H3 and 18 H1 strains was evaluated.

### Statistical analysis

To evaluate the immunogenicity of the rTET-NA vaccines, a multiple comparison analysis was performed using Tukey’s test to compare vaccine groups in terms of NAI titer production and to evaluate whether there was an adjuvant impact or a dose effect.

In the ferret challenge study, mean percent change in body weight graphs were created to show the pattern of the body weight loss from D44 to D57 per vaccine group in ferrets that were monitored up to D57 (*n* = 6 per group). The AUC of the body weight loss graph as well as the peak temperature rise were calculated, first individually, then per vaccine group. Bar charts were created to visualize these metrics per vaccine group. The AUC of the virus shedding was estimated per vaccine group using the trapezoidal rule.

To assess the association between anti-NA level and disease severity, a severity score was established based on a percentile approach. The individual severity score for each protection endpoint was defined by using the percentiles (25th, median and 75th) presented in Supplemental Table S1 (disease severity scoring). A combined severity score was then calculated per animal as the sum of the severity score of peak body weight loss, peak body temperature rise and the AUC of virus shedding. The combined severity score was calculated only for ferrets that had complete data for all three protection endpoints. Ferrets that had a combined symptom severity score above the 75th percentile of the distribution of the severity scores were considered as having severe disease. The comparison of NAI titers at D42 between groups was conducted using one-way ANOVA. The comparison was performed using log-transformed NAI titers to meet the normality assumption required for the use of the statistical model.

A binary outcome (severe or non-severe disease) logistic model was performed to evaluate the prediction of severity with NAI titer. In this model, the disease severity was defined as a dependent variable and the log-transformed NAI titer was included in the model as a predicted factor. The subsequent ROC curves generated were used to define cutoffs based on the maximum Youden’s index.

The AUC of the ROC curves were also estimated and used to evaluate the ability of the NAI titer to properly distinguish ferrets with severe disease from those with non-severe disease. An AUC of 0.5 means that the NAI titer is uninformative for the prediction of influenza severity. The closer the AUC is to 1, the better the discrimination power of the NAI titer.

All statistical tests were two-sided, and the nominal level of statistical significance was set to α = 0.05 for effect size estimates. Statistical analyses were conducted using SEG SAS v9.4^®^ (WISE environment) and R Statistical Software (R version 3.5.1 on RStudio^®^).

## Supplementary information

Supplementary information

## Data Availability

All data generated or analyzed during this study are included in this published article (and its supplementary information files).
