# Supplementary material for: Boosting neuraminidase immunity in the presence of hemagglutinin with the next generation of influenza vaccines
Source: NPJ Vaccines. 2024 Nov 19;9:228. doi: 10.1038/s41541-024-01011-x (PMC11577023; doi:10.1038/s41541-024-01011-x)
Supplement: Supplementary file 1 — Supplementary information [file 41541_2024_1011_MOESM1_ESM.pdf]

Supplementary materials

List of rHAs used in the antibody forensics assay in this study

H3 antibody forensics panel: A/URUGUAY/716/2007, A/VICTORIA/361/2011, A/HONGKONG/4801/2014, A/SINGAPORE/INFIMH-16-0019/2016, A/BRISBANE/1059/2017, A/ETHIOPIA/1877/2017, A/KENYA/105/2017, A/MISSOURI/37/2017, A/MIYAZAKI/89/2017, A/OSORNO/60580/2017, A/SAPPORO/46/2017, A/SHANGHAIXUHUI/1373/2017, A/SYDNEY/1093/2017, A/SYDNEY/1013/2017, A/AKSARAY/4048/2016, A/ALBANIA/7165/2016, A/ANKARA/4110/2016, A/BRETAGNE/2836/2016, A/BRISBANE/1009/2016, A/CALIFORNIA/168/2016, A/CHIBA/33/2016, A/CHRISTCHURCH/513/2016, A/GUANGXIQIXIN/328/2016, A/HAWAII/67/2016, A/JORDAN/J16420301NT/2016, A/KAWASAKI/142/2016, A/KHMELNITSKY/719/2016, A/LAOS/F2884/2016, A/LINKOU/0051/2016, A/LISBOA/NIEVA063/2016, A/MARTINIQUE/531/2016, A/MARYLAND/24/2016, A/MEKNES/168/2016, A/MICHIGAN/84/2016, A/PORTUGAL/MS68/2016, A/SAUDIARABIA/192150/2016, A/SHANDONGLAICHENG/1763/2016, A/SINGAPORE/GP2366/2016, A/TASMANIA/97/2016, A/TOWNSVILLE/51/2016.

H1 antibody forensics panel: A/CALIFORNIA/07/2009, A/BAYERN/69/2009, A/HONGKONG/34079/2009, A/HONGKONG/33597/2009, A/LVIV/N6/2009, A/MONTPELLIER/2051/2009, A/CHRISTCHURCH/16/2010, A/ANKARA/TR40/2011, A/ASTRAKHAN/1/2011, A/HONGKONG/3934/2011, A/GOTEBORG/1/2011, A/MEXICO/2208/2011, A/HONG/KONG/5659/2012, A/STOCKHOLM/25/2012, A/ISRAEL/Q504/2015, A/MICHIGAN/45/2015, A/HUNGARY/12/2016, A/BRATISLAVA/342/2016.

Table S1. Severity score and score definitions of the peak body weight loss, the peak of body temperature rise and the AUC for virus shedding.

**Severity score**	**Peak body weight loss (%)**	**Peak temperature rise (°C)**	**AUC virus shedding**
0 (Normal)	Weight increase or less than –3.42	<0.7	<8.375
1 (Mild)	[3.42–5.56]	[0.7–0.9]	[8.375–13]
2 (Marked)	[5.56–7.46]	[0.9–1.4]	[13–18.5]
3 (Severe)	>7.46	>1.4	>18.5

AUC, area under the curve

Figure S1. Immunogenicity of A/Singapore/INFIMH-16-0019/2016 N2 and A/Michigan/45/2015 N1 rTET-NA, LVNA and IIV preparations was tested in naïve mice. Female BALB/c mice (n=8 per group) were immunized twice intramuscularly and terminally bled 2 weeks after second immunization as shown in Figure 2A. Sera were collected two weeks after booster vaccination; sera pools from two animals were created and in turn tested via ELISA to assess NA-binding antibody titers using A/Singapore/INFIMH-16-0019/2016 N2 rTET-NA (A) or A/Michigan/45/2015 N1 rTET-NA (B) as coating antigens, respectively. ○ symbol represents EC_{50} antibody titers without AF03 addition and ■ represents groups with AF03 addition. The dashed line indicates the starting serum dilution used for testing. *p <0.05; ** p<0.01; *** p<0.001 (the red text/symbols are comparisons vs adjuvanted PBS/control). For comparisons between adjuvanted and non-adjuvanted matched dose and formulation, all were significant with p <0.001 (except for LVNA 0.2µg [p<0.05] and IIV 1 µg [p <0.01] A/Singapore/2016 N2). LLOD: Lower limit of detection

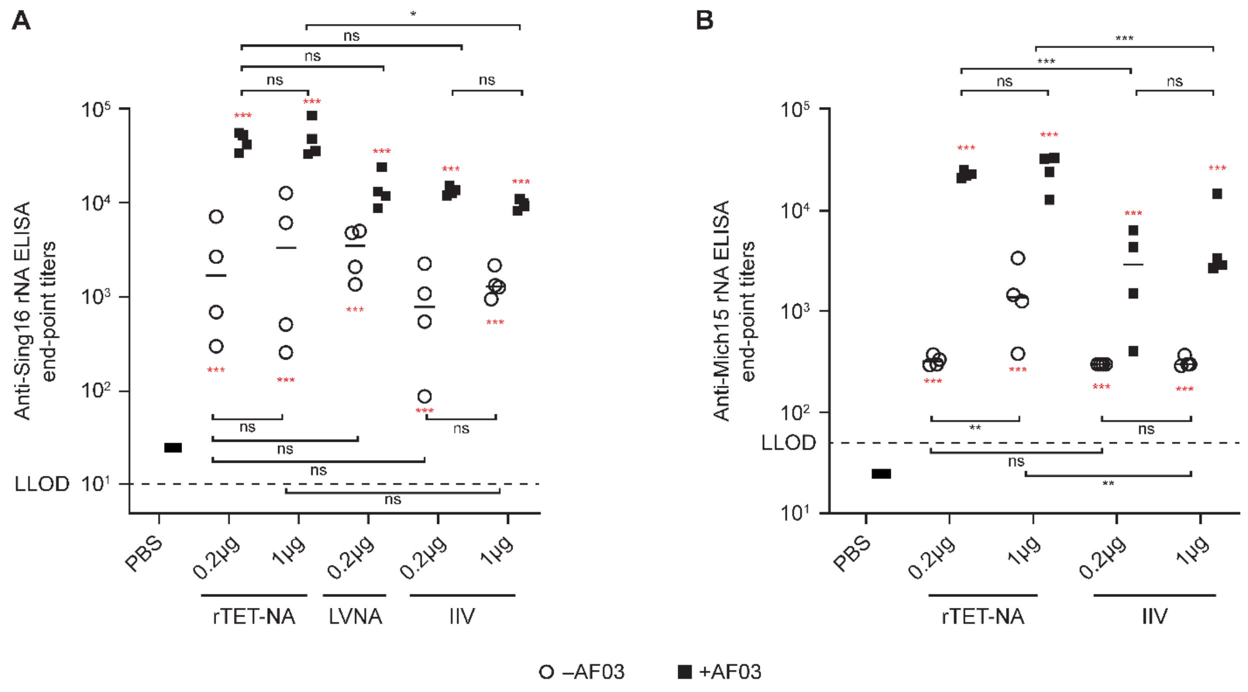

Figure S2. rTET-NA retains its immunogenicity following octavalent HA and NA vaccination in ferrets. NA-binding antibody titers against vaccinal N2, N1, B/Victoria-like and B/Yamagata-like strains were determined in sera from vaccinated ferrets three (3) weeks after each immunization via ELISA using corresponding rTET-NA as coating antigens. For all comparisons between 1 dose and 2 doses, all NA containing groups had similar or higher NA-binding antibody titers after 2 doses, while no difference or higher NA-binding titers were observed upon addition of 4x HA, statistical analysis pending. NT: Not tested. LLOD: Lower limit of detection

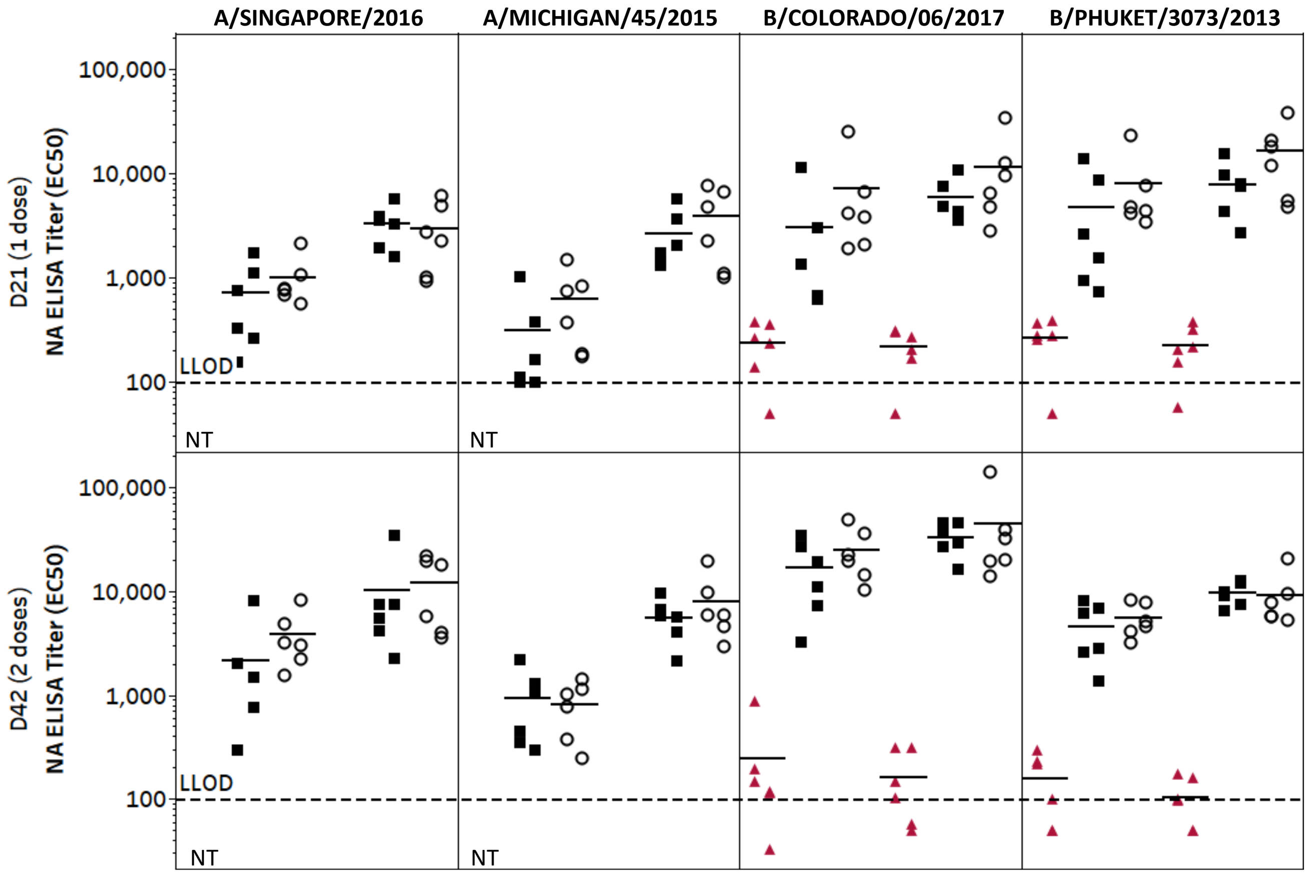

Figure S3. Post-vaccination NA-binding antibody titers are a correlate of protection in the ferret model. Pre-challenge PE09 anti-N2 NA-binding antibody titers were measured in sera collected three weeks after the second vaccination via ELISA using PE09 rTET-NA as coating antigen (A). ROC curve (B) and distribution of the pre-challenge NA ELISA titers according to severity score (C).

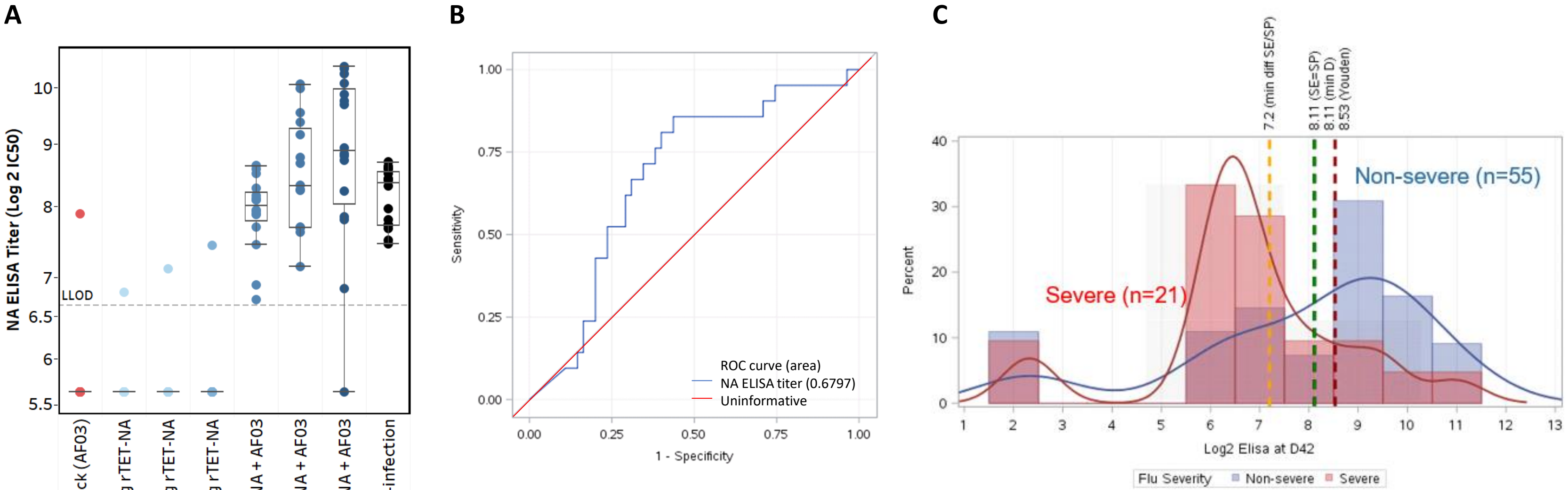

AUC name	AUC [95%CI]	*P*-value
NA ELISA titer	0.68 [0.55;0.81]	**0.0071(S)**
Uninformative	0.50 [0.50;0.50]	

Criterion	Criterion value	Cut-off value	Sensitivity	Specificity
Maximum Youden's index	0.42	8.53	85.71%	56.36%
